# A preliminary study of auditory mismatch response on the day of cochlear implant activation in children with hearing aids prior implantation

**DOI:** 10.1371/journal.pone.0210457

**Published:** 2019-01-07

**Authors:** Yuan Li, Min Shen, Mo Long

**Affiliations:** 1 China-Japan Friendship Hospital, Beijing, China; 2 China Rehabilitation Research Center for Hearing and Speech Impairment, Beijing, China; Center for Healthy Start Initiative, NIGERIA

## Abstract

**Objective:**

The study aimed to explore the characteristics of auditory mismatch response (MMR) in hearing-impaired children on the day when the cochlear implant (CI) was started (power-up) and the speech processor was programmed, and to investigate the effects of wearing hearing aids (HAs) before cochlear implantation on the early stage of postoperative auditory cortex plasticity, providing some demonstrative data for the objective evaluation of postoperative early auditory ability in children who underwent cochlear implantation.

**Methods:**

The participants were 34 children with profound sensorineural hearing loss, who underwent cochlear implantation. The classical passive Oddball paradigm was adopted, using a pair of vowels which only have different lexical tones. The standard stimulus was /a2/ and the devious stimulus was /a4/.

**Results:**

1) On the day of CI activation, the auditory MMR has been elicited in 30 children; the MMR incidence was 88%. 2) We observed both positive and negative auditory MMR waveforms. And logistic regression analysis showed that it was influenced by the age at cochlear implantation. 3) The duration with HA before surgery significantly influenced the MMR latency. The children with longer duration of HA use have much earlier latency of MMR. 4) There was a significant positive correlation between the age at HA use initiation and MMR amplitude. Earlier initial HA use was associated with smaller amplitude.

**Conclusions:**

MMR in response to Mandarin lexical tone can be recorded in most pediatric patients who had experience with HA on the day of CI power up. MMR is closely associated with the age at cochlear implantation, duration of HA use, and the age at HA use initiation. Hearing-impaired children should wear HA as early as possible and ensure consistent usage.

## Introduction

There are quite a few studies [1–5] regarding the effects of wearing HA before cochlear implantation on auditory and speech rehabilitation. However, most of these studies adopted subjective tests [6–9] and there has been no consensus regarding the significance of wearing HAs before surgery. This study aimed to examine Mandarin lexical tones’ discrimination of Mandarin-speaking children on the day of CI activation and the effects of HA use prior implantation by an indicator which was called MMRs, one of event-related potentials (ERPs). This will provide an objective evaluation of the postoperative auditory ability of children who underwent the electric cochlear implantation at a super-early stage and examine the effects of wearing HAs before surgery on postoperative early auditory cortex remodeling in these children.

## Materials and methods

### Participants

A total of 34 children (21 boys, 13 girls) with profound sensorineural hearing loss and an average aided sound field threshold of 60 dB HL (Hearing Level) or above were recruited. Seven patients had CI in the left ear, while 27 patients had CI in the right ear. Sixteen patients have electric CIs with the speech processor of Medel-Opus II, ten patients with Cochlear Freedom, three patients with N5, and five patients with AB Harmony respectively. The age at HA use initiation was 8–83 months (mean: 23.44 months) and the duration of HA use was 0.03–107 months (mean: 16.71 months). The children’s age at cochlear implantation ranged from 19 to 166 months (mean: 41.32 months). Eleven patients exhibited comorbid white matter abnormalities on cranial MRI, four patients exhibited comorbid large vestibular aqueduct syndrome (LVAS), and one patient exhibited comorbid Wardernberg syndrome. The mental test results of all 34 participants were normal. An auditory MMR test was performed after CI activation (on the same day). We obtained written informed consent from the parents or legal guardians of all the participants. The research protocol was approved by the Institutional Review Board of the China Rehabilitation Research Center for Hearing and Speech Impairment.

### Stimuli

The passive Oddball paradigm was used and the probabilities of standard and deviant stimuli were 85% and 15%, respectively. The lexical tones consisted of /a2/ (Tone 2, the high rising tone) and /a4/ (Tone 4, the high falling tone). The standard stimulus was /a2/ and the deviant stimulus was /a4/. The lexical tone stimuli were produced by a female native Mandarin speaker and recorded at a 44.1 kHz sampling rate with Computer Speech Lab (CSL) and were normalized to 200 ms with Praat (http://www.fon.hum.uva.nl/praat/).

### Procedure

Participants were seated in an acoustically and electromagnetically shielded chamber and listened to the sound stimuli from a loudspeaker (Hi-Vi X4) located 1.5m in front of their heads, while facing a 17-inch monitor at a distance of 1.2m. The loudspeaker was located behind the monitor, the distance between the loudspeaker and the monitor was 0.3m. The stimuli were presented over one loudspeaker at 70 dB SPL (Sound Pressure Level). The CI user underwent free-field stimulation through the device's microphone. The inter-stimulus interval (ISI) was 450–500ms. The stimuli were presented in a pseudorandom sequence with at least three standard stimuli presented before a deviant stimulus. The entire experiment lasted approximately 13 minutes.

### Electroencephalographic (EEG) recording and analysis

Electroencephalographic (EEG) data were recorded using SynAmps 2 amplifier (NeuroScan 4.5, Charlotte, NC, U.S.) with a cap carrying 64 Ag/AgCl electrodes placed on the scalp at the standard locations according to the extended international 10–20 system. Vertical electrooculography (EOG) was recorded using bipolar channel placed above and below the left eye, and horizontal EOG was recorded using bipolar channel placed lateral to the outer canthi of both eyes. All electrodes were referenced to the tip of the nose. The ground electrode was situated on the participant's forehead. The impedance was below 5 KΩ throughout the recording. The EEG was recorded at a sampling frequency of 1000 Hz with a band-pass filter setting from 0.1 to 100 Hz.

Offline analysis was performed using the recorded raw EEG data. The sequential steps were: removal of portions with significant drift and interference, band-pass filtering of 0.1–30.0 Hz (filter), removal of EOG artifacts, epoch, baseline correction, artifact rejection, averaging and subtraction. The waveform of the standard stimulus was subtracted from the waveform of the deviant stimulus. There were three types of tag for standard stimuli (“22”, “20” and “2” respectively) before the deviant stimulus (“24”). The first type of standards (“22”) was the one followed by a deviant stimulus (“24”). And the second type of standards (“20”) was the one followed by the first type. The third type of standards was the one preceding the second type. So the number of deviants and the first/second type of standards were the same. The responses elicited by the first type of standard stimuli were used in the subtraction to obtain MMR. The responses elicited by the other two types of standard stimuli were not included in the MMR analysis but the responses elicited by the second type standard stimuli (“20”) were averaged and compared with the average of the first type of standard stimuli (“22”) as a way to examine consistency and accuracy. The validity of MMR was referenced to the polarity at the right, left mastoid or Oz and brain topographic map. The maximum difference wave (maximum negative wave or maximum positive wave) between 100 and 300 ms was confirmed as MMR. The latency and amplitude at Fz were analyzed. Since we did not find the artifacts of CI in the majority of patients, and some artifacts were not obvious in two children and did not influence the MMR detection, we did not remove some subtle CI artifacts if there were by EEGLAB.

Data was analyzed by SPSS 19.0 software. Normality testing and variance homogeneity testing were performed. Data that conformed to these conditions were used for independent samples t-test. Also, chi-square test, bivariate simple correlation analysis and partial correlation analysis were performed. T-test was performed to explore whether there was significant difference on the age at HA use initiation, age at cochlear implantation, latency and amplitude of MMR between two groups of children with different duration of HA use. Chi-square test was performed to explore whether there was difference on the number or incidence of MMR between two groups of children with different duration of HA use, also difference on the incidence of negative and positive waves between two groups of children with different age at cochlear implantation. Bivariate simple correlation analysis was performed to explore the relationship between ages at cochlear implantation, age at HA use initiation and the duration of HA use. Furthermore, partial correlation analysis was performed to explore the relationship between ages at HA use initiation and the latency, amplitude of MMR.

## Results

### Incidence of MMR

**Fig 1:**
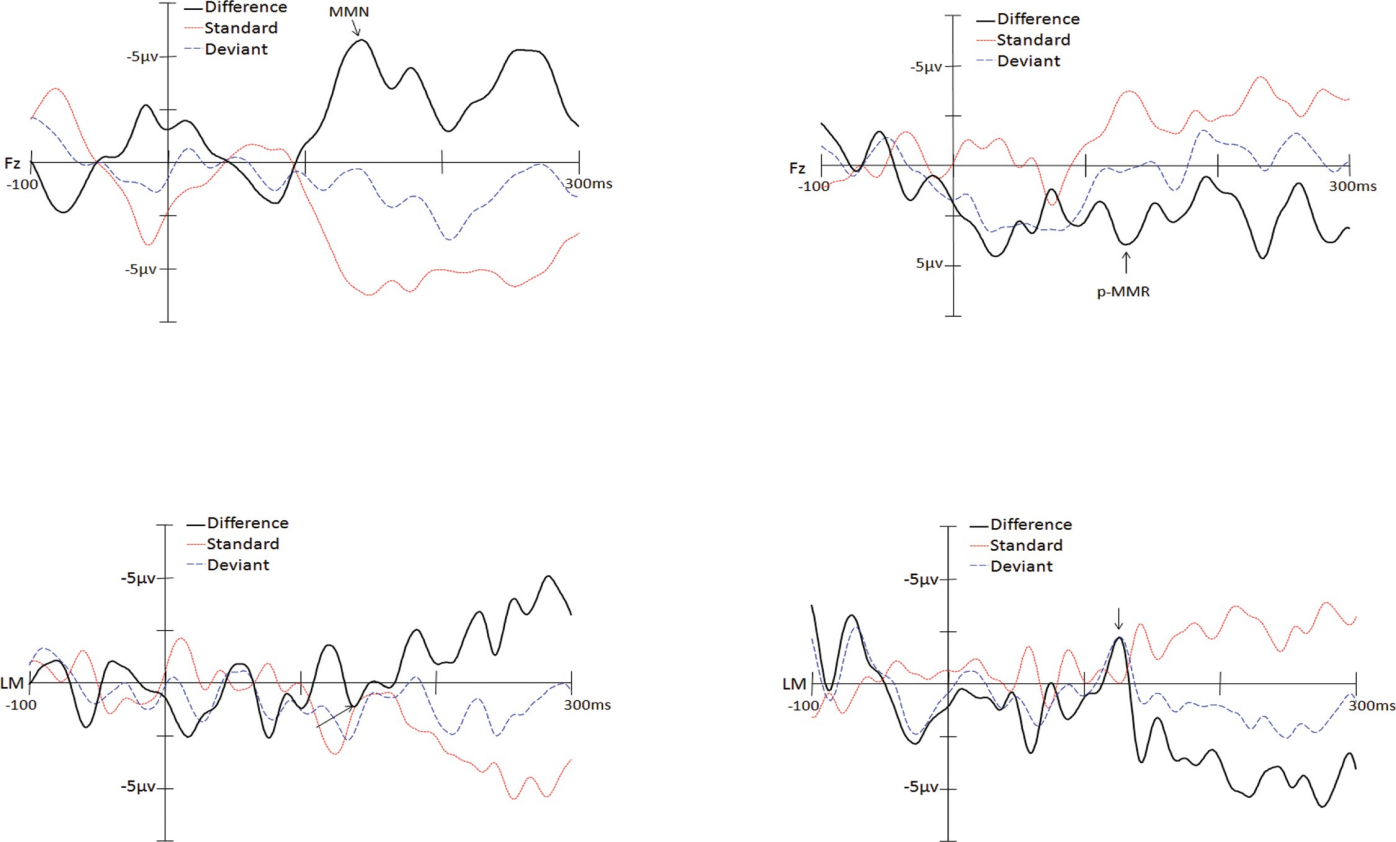
a) Mismatch negativity of Mandarin lexical tone discrimination in a child with CI; b) Positive-mismatch response of Mandarin lexical tone discrimination in a child with CI. (Deviant: deviant stimulus; Standard: standard stimulus; Difference: difference wave; p: positive wave; N: negative wave).

On the day of CI activation, auditory MMR was elicited in 30 of 34 children (88%) who underwent cochlear implantation surgery (Tables 1 and 2). Among these 30 patients, 15 (50%) showed positive mismatch responses (p-MMR) and 15 (50%) showed mismatch negativity (MMN). Fig 1 showed typical auditory MMN (a) and p-MMR (b) respectively in two children.

**Table 1:** Incidence of MMR in children with CI who wore HAs for different duration.

Group	*n*	Not elicited (%/*n*)	Elicited (%/*n*)	
A	10	30 (3)	70 (7)	χ^2^ = 4.10
B	24	4 (1)	96 (23)	p<0.05

(Group A: Duration of HA use <6 months; Group B: Duration of HA use ≥6 months)

**Table 2:** Basic information of four patients without MMR.

**No.**	**Gender**	**Age at cochlear implantation(months)**	**Age at HA use initiation (months)**	**Duration of HA** **use(months)**	**Comorbidity**
	M	166	59	107	No
	F	27	23	4	LVAS, slightly small cochlea
	F	21	8	<2	No
	F	24	15	<1	No

HA: hearing aid; LVAS: large vestibular aqueduct syndrome.

### Analysis of influencing factors on the auditory MMR waveform

The polarity of the auditory MMR (two levels: positive and negative) was used as a dependent variable; and the age at HA use initiation, the duration of HA use, the age at cochlear implantation were used as independent variables for logistic regression analysis (Forward). The results found that R^2^ of the model was 0.268 (Table 3). The main influencing factor for waveform direction was the age at implantation (χ^2^ = 6.72, p<0.05). Children who were implanted with the electric cochlear mostly below the age of 2 years old exhibited negative waves (Table 4).

**Table 3:** Analysis of influencing factors on the waveform of auditory MMR.

Influencing factors	β	S.E.	Wald	*P*	Odds Ratio
Age at cochlear implantation (below or above 2 years old)	2.51	1.16	4.69	0.03	12.25

**Table 4:** Auditory MMR characteristics in children of different ages at cochlear implantation.

Age at cochlear implantation	*n*	MMN	p-MMR	
<2 years old	8	7	1	χ^2^ = 6.72 p<0.05
≥2 years old	22	8	14	

### Relationship between auditory MMR and duration of HA use

The 30 children were divided into two groups based on the duration of HA use: HA use for <6 months (Group A) and HA use for ≥6 months (Group B). There was no significant difference between the two groups regarding the age at HA use initiation and the age at cochlear implantation [*t*(1,28) = 0.80, p>0.05; and *t*(1,28) = 1.86, p>0.05, respectively]. Chi-square test revealed that the incidence of auditory MMR in children of Group A was significantly lower than which in children of Group B (χ^2^ = 4.10, p<0.05). There were no significant differences on the incidence of negative and positive waves between Groups A and B (χ^2^ = 1.72, p>0.05). Independent samples t-test showed that there was significant difference on the latency of MMR between the two groups. The latency in children of Group B was significantly earlier than which in children of Group A (t = 2.13, p<0.05). The difference on amplitude between both groups was not significant (t = 0.36, p >0.05) (Table 5).

**Table 5:** MMR characteristics in cochlear implanted children with different duration of HA use.

Group	n	Latency	Absolute value of the amplitude (μV)	MMN (%/*n*)	p-MMR (%/*n*)
A	7	227.29±36.30	5.42±3.06	71 (5/7)	29 (2/7)
B	23	180.96±53.66	4.90±3.41	43.4 (10/23)	56.5 (13/23)
		t = 2.13	t = 0.36	χ^2^ = 1.72	
		p <0.05	p >0.05	p>0.05	

### Relationship between auditory MMR and the age at HA use initiation

Bivariate simple correlation analysis found that the age at HA use initiation(X) significantly positively correlated with the duration of HA use (Y) and the age at cochlear implantation (Z) (*r*_XY_ = 0.59, p<0.01; *r*_XZ_ = 0.90, p<0.001; *r*_YZ_ = 0.89, p<0.001). So we further used the duration of HA use as a control variable for partial correlation analysis and found that the age at HA use initiation had a significant positive correlation with auditory MMR amplitude (*r* = 0.39, p<0.05). When the age at cochlear implantation was used as a control variable for partial correlation analysis, we found that the age at HA use initiation had a significant positive correlation with MMR amplitude (*r* = 0.40, p<0.05). Table 6 showed the results.

**Table 6:** Partial correlation analysis of the age at HA use initiation and MMR latency, amplitude.

Control variable		Latency	Amplitude (absolute value)
Duration of HA use (Y)	Age at HA use initiation(X)	r = -0.01	r = 0.39
		p >0.05	p <0.05
Age at cochlear implantation(Z)	Age at HA use initiation(X)	r = 0.11	r = 0.40
		p >0.05	p <0.05

## Discussion

### Auditory MMR may be used as an objective evaluation indicator for auditory abilities

MMR is one component of ERPs and can be an indicator of automatic discrimination towards differences between many kinds of stimuli. Näätänen et al. have first reported the MMN in 1978 [10–11]. MMN typically presents as a negative difference wave between the deviant and standard stimuli, which appears at around 100–250 ms in adults. Recently, in addition to the mismatch negativity in infants and children that are similar to those observed in adults, many studies have reported p-MMRs [12–20] in children. And they are called MMR together in the studies. Some studies [10–20] have showed that MMR can reflect the basic mechanisms of auditory discrimination and processing. Also, MMR can trace the effects of natural maturation and interferences on central auditory processing. In addition, some researches [21–23] have shown that auditory MMR can provide early diagnosis information for the identification of central auditory processing disorder, developmental language disorder, and dyslexia. The comparison of the waveforms, latency and amplitude of MMR provides unparalleled advantages in implementing auditory and speech ability assessment and in exploring the plasticity of central auditory system [24–34]. Currently, this is the only objective evaluation approach available for pre-attentive auditory discrimination. There are a few studies on the effects of HA use before cochlear implantation on auditory and speech rehabilitation. However, most of them have adopted subjective tests [1–5]. Therefore, this study adopted auditory MMR as an objective evaluation indicator, and was the first to use Mandarin lexical tones as the sound stimuli to assess the post-operative auditory discrimination ability of pediatric patients with severe and profound hearing loss, at a super-early stage after cochlear implantation. The results can help to examine the effects of wearing HAs before surgery on auditory cortex reconstruction in children at an early stage after the electric cochlear implantation.

There are a lot of auditory stimuli used in the MMR paradigm, such as different vowels, consonants, lexical tones, pure tones, and lexical tones are much easier to be discriminated. Some researchers suggested that the stimulus should be not very difficult to discriminate for the children with CI in the clinical practice [35]. Tone 2 (the increasing curve) and tone 4 (the falling curve) have the greatest difference compared with any other two lexical tones in Mandarin Chinese. It seems that it was the easiest sound stimulus pair to be discriminated. And the high incidence of the MMR showed that it was appropriate to investigate the MMR of cochlear implanted children on the day when the CI was activated. And we conclude that the MMR test using the stimuli can be used as an objective evaluation method on auditory abilities. However, the stimuli have two shortcomings as followed. Firstly, it may be just appropriate for children who can get access to or have some experiences on tonal languages, such as mandarin Chinese, Cantonese. Secondly, the tone pair is so easy to be discriminated that it should be used to explore the preliminary auditory discrimination ability for hearing impaired children (including children with HA or CI). And we should use much more difficult sound pairs and more kinds of stimuli to investigate subtle or complicated auditory discriminative ability.

### Incidence of auditory MMR

MMR is elicited in the cortex and associated with the amount of acoustic information that can be discriminated by the listener. The use of MMR can help to detect retarded speech development caused by auditory cortex dysplasia extremely early. The present study recording ERP and analyzing the MMR on the day when CI was activated aimed to examine the pre-attentive auditory discrimination ability of children with CI as early as possible and explore the effects of HA use before the surgery. The incidence of auditory MMR reflects the auditory perception of the auditory cortex and automatic discriminative processing on Mandarin lexical tones by the CI at a super-early stage.

There have been a few studies that aimed to examine auditory MMR in children on the day of CI activation. Liang et al [32] have investigated the MMN to frequency difference of pure tones in children with prelingual hearing loss who underwent cochlear implantation. And the result has found that the incidence of MMN on the day when the CI power up in the study was zero. Chen et al. [33] have found that the auditory MMR incidence was 50% in six patients with post-lingual hearing loss on the day when CI was activated. This study showed that the incidence of auditory MMR in pediatric patients was 88% (30/34) on the day when the CI was activated. It was much higher than which in previous studies. This may be associated with the following aspects. Firstly, this study used Mandarin lexical tones as sound stimuli, tone 2 (the increasing curve) and tone 4 (the falling curve) which have the greatest difference compared with any other two lexical tones in Mandarin Chinese. Notably, Mandarin lexical tones have supra-segmental features and thus can be much easier to perceive and discriminate, compared with frequency differences on pure tones or vowel differences that were commonly used in previous studies. Secondly, the ERP data was recorded when subjects were awake in this study. There has been no consensus regarding whether sleep status affects MMR incidence or not. However, a reportedly low incidence may be related to the use of 10% chloral hydrate sedation [32] in the EEG recording. Thirdly, this may be related to the HA use experience or HA fitting of pediatric patients before surgery. In the literature, the HA use status of children with prelingual hearing loss who showed no elicited auditory MMR, was not clear. We can only find that the age at cochlear implantation was 14–73 months and most children used sign language or gestures [32]. However, in the current study, most children wore HA before surgery and for a longer period. With the exception of a single 14-years-old patient, the remaining children were implanted with the electric cochlear at younger age and could generally speak simple words or even sentences. Among the four patients without measurable MMR, one showed comorbid inner ear deformities and wore HAs for only 4 months, two patients had worn HAs for less than 2 months and had been deprived from sound stimuli for a long time (having stopped wearing HA for more than 8 months, and one child underwent implantation at 14 years old. Although the latter had worn HAs for nearly 8 years, the auditory compensation results were poor and the patient mainly used sign language for communication. And the patient’s auditory performance required further follow-up. According to the provided data, especially 4 cases without measurable MMR, it might be noticed that using HA and hearing experience affect MMR more than just wearing HA. We will discuss some factors influencing the MMR, such as the duration of HA use, the time at HA use initiation, age at cochlear implantation in the following part. And we should take more factors into account in the further study, such as HA fitting effect, hearing experience and communication environment.

### Characteristics of Auditory MMR on the day of CI activation

#### Waveform characteristics and influencing factors

There is a general consensus that adults usually exhibit the negative wave, the MMN in the MMR studies [11]. However, the MMR research involving infants and preschool children sometimes exhibit negative wave with a longer time window or the peak latency occurs at a later time point, which may be delayed to 400ms. And some studies [13–14] have found that MMR in infants is usually p-MMR at 200–450 ms, rather than MMN. Moreover, p-MMR was found in children with normal hearing [15–20]. This shows that MMR may undergo a developmental trace from positive slow waves to adult-like, rapid negative waves. With increasing age and development of the auditory central nervous system, p-MMR is weakened while adult-like MMN becomes apparent [16].

In this study, 15 of 30 children exhibit p-MMR, whereas the remaining 15 exhibit MMN. This is consistent with previous studies [12–20] and provides demonstrative data for positive and negative MMR simultaneously existing in preschool children. The study by Lee et al. [16] has found that both Mandarin vowel differences and lexical tone differences can elicit auditory MMR in 4–6-years-old children with normal hearing. A stimuli pair with greater differences can elicit adult-like MMN, while a stimuli pair with smaller differences can elicit p-MMR. Consonant differences only elicit p-MMR in 4–6-years-old children with normal hearing, which may be due to smaller differences between the standard stimuli and the devious stimuli.

The polarity of MMR and the incidence of p-MMR or MMN in children may be related to the following two aspects: 1) Age at cochlear implantation or maturity level of the auditory central nervous system. The development of the auditory nervous system and the chronological age of the participants will significantly affect the results of electrophysiological performance [17]. MMR is elicited apparently in children who wear HA early, or in children who have better effects when HA is worn, and can be manifested as different polarity. 2) Differences between stimuli. The magnitude of the difference between the standard and deviant stimuli may induce different polarity of the difference wave [12, 15–16, 18–19]. Additionally, this study showed that the age at cochlear implantation was one influencing factor of the MMR polarity in children. The incidence of MMN is much higher in children implanted before the age of 2 years old, compared with which in children implanted after the age of 2 years old. The specific reasons require further exploration. However, regardless of positive or negative MMR that was elicited, the results indicated that the auditory central nervous system of most pediatric patients with a history of HA use have pretty good automatic discrimination capacity on Mandarin lexical tones on the day when the CI is activated. And this provides demonstrative evidence on the integrity of the auditory pathway and robust plasticity of auditory central nervous system.

#### The Latency and amplitude characteristics of MMR

Auditory MMR latency reflects the timing in which responses to the stimuli, while amplitude usually reflects the magnitude of psychological resources consumed during information processing. The amplitude shows a strong positive correlation with the number of activated neurons and activation intensity [36]. The neural efficiency hypothesis [37] suggests that the working efficiency of the brain is manifested in two aspects: activation of brain regions related to the task, as well as non-activation of brain regions that are not related to the task. ERP latency and amplitude reflect brain activity levels, as reduced amplitude and shortened latency show that the working efficiency of the brain is higher. The present study found that on the day of CI activation, auditory MMR is affected by the duration of HA use in children. Pediatric patients who have worn HAs for 6 months or more exhibited significantly earlier auditory MMR latency. Additionally, auditory MMR amplitude was closely associated with the age at HA use initiation. When the duration of HA use and the age at cochlear implantation were fixed, children who began to wear HAs earlier exhibited smaller auditory MMR amplitude to Mandarin lexical tones. The reverse situation was also observed. The results of this study conform to the neural efficiency hypothesis, i.e. children who have worn HAs for a longer period, or who began wearing HAs at an earlier age, have a significantly higher brain working efficiency in processing Mandarin lexical tonal signals after cochlear implantation surgery. We hypothesize that wearing HAs continuously proceeds auditory stimuli that are conducive to the development of the auditory nervous system. That is, HA use can prevent the children being deprived from sound stimuli. And wearing HAs earlier can prevent developmental retarding or potential atrophy of the auditory nervous system. Further, auditory MMR is intimately associated with preoperative auditory and language experience: longer exposure to a language environment is associated with more abundant cumulative auditory and language experience. This is an important foundation for the reconstruction of the auditory central nervous system.

## Conclusions

In summary, our study adopted the auditory MMR to examine the objective lexical tone discrimination responses on the day when the CI is activated in most pediatric patients with a history of wearing HAs. MMR is intimately associated with the duration of HA use and the age at HA use initiation. For patients with profound sensorineural hearing loss, even if auditory compensation results are limited, HAs should also be selected and worn as early as possible before cochlear implantation. This is extremely important for the development and reconstruction of the auditory pathway and the auditory nervous system, as well as for the prevention of auditory deprivation. In addition, this will result in robust postoperative reconstruction of the auditory central nervous system.

However, with advocacy and education for the prevention and treatment of hearing impairment in China becoming deeper, pediatric patients generally have a history of wearing HAs before cochlear implantation surgery. Thus, there are very few hearing-impaired children who do not have a history of wearing HAs before surgery. In the future, we will design more types of stimuli, perform follow-up at different stages after the surgery, and combine with subjective assessment methods, such as speech audiometry and questionnaires, to further examine the auditory MMR and plasticity of the auditory cortex in children who undergo cochlear implantation.

## Supporting information

S1 File Dataset (SAV)
